# Contributions of Incidence and Persistence to the Prevalence of Childhood Obesity during the Emerging Epidemic in Denmark

**DOI:** 10.1371/journal.pone.0042521

**Published:** 2012-08-10

**Authors:** Lise Geisler Andersen, Jennifer L. Baker, Thorkild I. A. Sørensen

**Affiliations:** Institute of Preventive Medicine, Copenhagen University Hospital, Copenhagen, Denmark; John Hopkins Bloomberg School of Public Health, United States of America

## Abstract

**Background:**

Prevalence of obesity is the result of preceding incidence of newly developed obesity and persistence of obesity. We investigated whether increasing incidence and/or persistence during childhood drove the prevalence of childhood obesity during the emerging epidemic.

**Methods:**

Height and weight were measured at ages 7 and 13 years in 192,992 Danish school children born 1930–1969. Trends in the incidence (proportion obese at 13 years among those not obese at 7 years) and persistence (proportion obese at 13 years among those obese at 7 years) across birth cohort periods (1930–41 with low stable prevalence of obesity, 1942–51 with increasing prevalence, 1952–69 with the higher, but stable prevalence) were investigated. Logistic regression was used to examine the associations between BMI at 7 years as a continuous trait, allowing interactions with the birth cohorts, and occurrence of obesity at 13 years.

**Results:**

The prevalence of obesity was similar at 7 and 13 years and increased across birth cohorts in boys from around 0.1% to 0.5% and in girls from around 0.3% to 0.7%. The incidence of obesity between ages 7 and 13 years increased from 0.15% to 0.35% in boys and from 0.20% to 0.44% in girls. The persistence increased from 28.6% to 41.4% in boys and from 16.4% to 31.0% in girls. Despite a decrease over time, the remission of obesity occurred in >60% of obese children in the last birth cohort. However, the odds ratios of obesity at age 13 years in relation to the full range of BMI at 7 years remained unchanged across the birth cohort periods.

**Conclusions/Significance:**

The development of the obesity epidemic in children was due to an increase in both incidence and persistence of obesity. Contrary to prevailing expectations, a large, although declining, proportion of children obese at an early age underwent remission during childhood.

## Introduction

The prevalence and severity of obesity among children and adolescents has increased worldwide during the last century [1]–[3]. It is now at epidemic levels and recognized as a serious public health problem [4]. Results from analyses of Copenhagen school children [5], [6] and Danish draftees showed that the obesity epidemic evolved in phases of increasing and stable prevalence [3], [7], [8]; the prevalence of obesity increased steeply among those born from 1942 to around 1951, then leveled off and remained stable for about two decades, when a second even steeper rise began.

Although obesity is considered the upper range of a continuous trait, usually measured as body mass index (BMI; weight(kg)/height(m)^2^), its occurrence is described by the prevalence of individuals in the population who, at a certain point in time, exceed a set level of the trait, which in children may depend on their gender and age [9]. Prevalence is a composite epidemiologic measure that needs to be broken down into its components to be properly interpreted. In a stable population with a constant low prevalence of the condition, the prevalence is approximately equal to the incidence rate times the mean duration of illness [10]. Applying this relation to the childhood obesity epidemic, it may be considered as due to an increased number of children developing obesity and/or an increase in the duration of the condition. An increasing duration of obesity means a decreasing number of children lose weight and become non-obese before the point in time when the prevalence is assessed.

Some studies have reported on the incidence of overweight and obesity among children [11]–[14]. The persistence of childhood obesity also has been quantified in a number of longitudinal studies [11]–[13], [15]–[18] which report a two- to ten-fold increased risk of obese children remaining obese into adulthood, corresponding to a persistence of 20–80%. None of these studies, however, has addressed trends across time in the incidence and persistence and their relationships to the trend in the prevalence of obesity during the development of the epidemic.

We investigated whether an increasing incidence and/or an increasing persistence of obesity from 7 to 13 years of age contributed to the first steep rise in the prevalence of obesity. Associations across the entire BMI distribution as well as with the occurrence of obesity were examined.

## Methods

### Study Design and Study Population

To address the aim of the study, longitudinal analysis of repeatedly measured BMI in several cohorts from before, during and after the emergence of the obesity epidemic is required.

Data allowing such analysis was obtained from The Copenhagen School Health Records Register (CSHRR) [5], which contains computerized information on children who ever attended schools in the Copenhagen municipality and were born in 1930 through 1989. Until 1983 the children underwent annual health examinations by doctors or nurses, which included measurements of height (without shoes) and weight (naked or in underwear only). Only a fraction of the children were measured before 7 years or after 13 years of age. From 1983, the law was changed and children had health examinations only at school entry or if they had special health needs. Consequently, the number of children who were measured in their 13^th^ year of life declined just before the birth year 1970 [5].

To attain the longest age span during childhood without losing information and introducing possible selection bias, we focused on the weight status transition from 7 to 13 years of age. Thus, children eligible for this study were children who were born between 1930 and 1969 and had information available on age, sex and measurements of height (cm) and weight (kg) at 7 and 13 years of age.

### Ethics Statement

The present analysis was conducted on anonymous data and it was approved by the Danish Data Protection Agency (*Datatilsynet*). According to the Danish Act of Processing of Personal data (*Persondataloven*) [19], informed consent is not required for register-based research of pre-existing personal data.

### Definition of Variables

The body weight (kg) and height (m) measured around ages 7 and 13 years were used for calculation of BMI (kg/m^2^). Exclusions of outliers prior to the analyses are described in the Supplementary note S1. Age 7 years was defined as 84 to 89 months, 7.5 years as 90 to 95 months, 13 years as 156 to 161 months and 13.5 years as 162 to 167 months. Children were classified as obese if their BMI exceeded the age- and sex-specific cut-off percentiles defined by the International Obesity Task Force (IOTF) [9]. These cut-off values were 20.63, 21.09, 26.84, and 27.25 kg/m^2^ for boys at age 7, 7.5, 13, and 13.5 years. Among girls, the respective values were 20.51, 21.01, 27.76, and 28.20 kg/m^2^ [9]. To assess secular trends in prevalence, incidence and persistence, the population was divided in birth cohorts 1930–41, 1942–51 and 1952–69, which corresponded to periods in which the prevalence of obesity among Danish young men examined at the draft boards was low and stable, steeply increasing, and then stable at the higher level, respectively [3], [7]. A similar pattern has been observed among boys in the present study population [8].

### Statistical Analyses

All analyses were conducted with SAS, version 9.1 (SAS Institute Inc., Cary, NC, USA).

Boys and girls were analyzed separately.

The correlation between BMI at 7 years and BMI at 13 years in each of the birth cohort periods was investigated with scatter plots and by linear regression analysis. A potential two-way interaction between BMI at 7 years (included as a continuous variable) and birth cohort (included as a categorical variable) on BMI at 13 years was assessed by the Wald test.

Trends in the upper tail of BMI at 7 and 13 years in accordance with the definitions of obesity were investigated. The *prevalence proportion* at ages 7 and 13 years was calculated as the percentage of all children who were obese at the given age. The relation between the obesity cut-off point and the distributions of BMI at 7 and 13 years of children who became obese or remitted from obesity was visualized with the scatter plots. The *incidence proportion* was calculated as the percentage of non-obese children who developed obesity from 7 to 13 years of age. The *persistence proportion* was calculated as the percentage of those obese at 7 years who were still obese at age 13 years. The *remission proportion* is equal to 1 minus the persistence proportion. The contribution from persistence, the *persistence contribution*, to the prevalence of obesity at age 13 years was defined as the percentage of those obese at age 13 years who were already obese at 7 years. The complementary *incidence contribution* was defined as the percentage of those obese at age 13 years who were non-obese at age 7 years. The latter two proportions sum to 1.00. Potential linear trends in these proportions were assessed by the Cochran-Armitage chi square test for trends [20], in which the three periods of birth cohorts were assigned equidistant scores.

The odds ratio (OR) of being obese at 13 years of age (yes/no) given BMI at 7 years across its full range and birth cohort periods was investigated by logistic regression. Potential two-way interactions between BMI at 7 years (included either as a continuous variable or categorized as: <14, ≥14–<15, ≥15–<16 (reference), ≥16–<17, ≥17–<18, ≥18–<19, ≥19–<20, ≥20 kg/m^2^ ) and birth cohort periods (included as a categorical variable) on obesity at 13 years were assessed by the Wald test. Linearity between BMI at 7 years and the odds of obesity at 13 years was assessed in sex- and cohort-period specific strata by estimating the OR in the same categories of BMI at 7 years and by graphing the OR values on a logarithmic scale. The analyses were adjusted for the age in months around 7 years.

## Results

Of 240,246 children who were born from 1930–69 and attended school in Copenhagen, 192,992 children (80.3%) had information on BMI at 7 and 13 years available. The sample included 96,849 boys and 96,143 girls, of whom 291 boys and 507 girls were obese at 7 years and 369 boys and 438 girls were obese at 13 years of age (Table S1 and Table S2).

### BMI at 7 Years and BMI at 13 Years of Age as Continuous Traits

**Figure 1:**
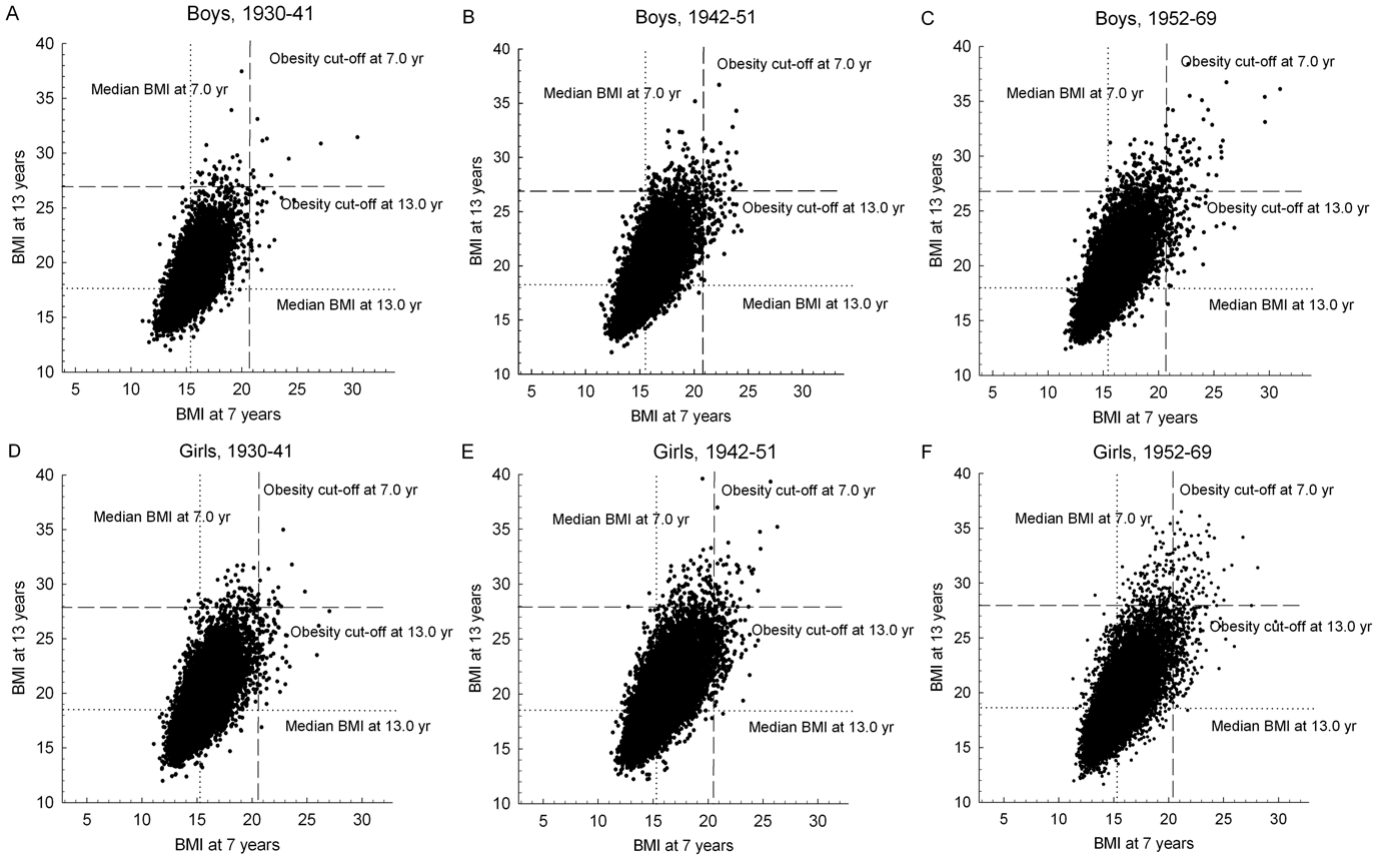
Correlation between BMI at 7 years and BMI at 13 years. The figure shows the correlation between BMI at 7 years and BMI at 13 years in each birth cohort among 96 848 boys (upper panels) and 96 106 girls (lower panels) in the Copenhagen School Health Records Register. Children with a BMI at age 7 years <10.5 (n = 38) were not included. Obesity was defined by International Obesity Task Force criteria. The dotted vertical and horizontal lines represent the cut-offs for obesity at 7.0 and 13.0 years respectively. For children aged 90 to 95 months and 162 to 167 months the cut-offs for 7.5 and 13.5 years respectively were used to define obesity. The four quadrates represent never-obese (lower left quadrant), incident obese (upper left quadrant), persistently obese children (upper right quadrant) and children who remitted from obesity (lower right quadrant) from 7 to 13 years of age.

The median BMI at age 7 years was 15.44 kg/m^2^ among boys and 15.31 kg/m^2^ among girls. The respective values at 13 years were 18.06 kg/m^2^ and 18.67 kg/m^2^. The median BMI at 7 and 13 years among boys and girls showed only small fluctuations across the three birth cohort periods (Figure 1). Among boys and girls in each birth cohort period, BMI at 7 and 13 years were, as expected, positively correlated (Figure 1); for each unit increase in BMI at 7 years, BMI at 13 years increased in the various gender and birth cohorts by 1.17 to 1.25 units. The strength of the association increased across the birth cohort periods (Table 1), as indicated by a statistically significant interaction between BMI at 7 years and birth cohort in their relation to BMI at 13 years in both boys (p<0.0001) and girls (*p* = 0.0028). The increase across birth cohort periods in the association between BMI at age 7 and 13 years was slightly stronger among boys than it was among girls (*p*−*_interaction_* = 0.0002).

**Table 1:** BMI at 13 years as a function of BMI at 7 years by sex and birth cohort^a^.

			Birth cohort			
	1930–41		1942–51		1952–69	
Sex	β-Coefficient^b^	95% CI	β-Coefficient^a^	95% CI	β-Coefficient^a^	95% CI
**Boys**	1.17	1.15, 1.18	1.24	1.22, 1.25	1.25	1.24, 1.26
**Girls**	1.19	1.18, 1.21	1.20	1.19, 1.21	1.23	1.22, 1.24

Abbreviations: CI, confidence interval.

a Children with a BMI at age 7 years <10.5 (n = 38) were not included.

b The estimate for the increase in BMI units at 13 years (kg/m^2^) per BMI unit increase at 7 years (kg/m^2^) is derived from a model including the main effect of BMI at 7 years, sex, cohort, age at 7 years in months and two- and three-way interactions between sex, birth cohort and BMI at 7 years.

### Trends in Prevalence, Incidence, Persistence and Remission Proportions

**Figure 2:**
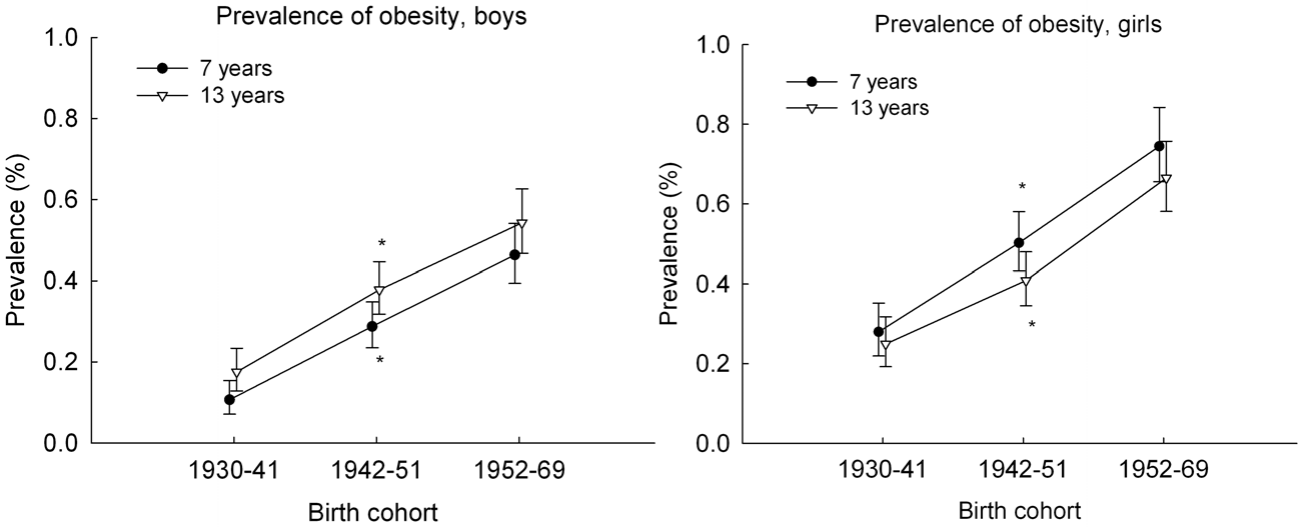
Trends in the prevalence proportion of obesity. The figure shows trends in the prevalence proportion of obesity at age 7 and 13 years (percent with 95% confidence intervals) by birth cohort (1930–1969) among 96 849 boys (left panel) and 96 143 girls (right panel) in the Copenhagen School Health Records Register. Note that the range on the y-axis is truncated at 1%. Obesity was defined by International Obesity Task Force criteria. * *p*_linear_ <0.001.

The prevalence of obesity at 7 and 13 years increased linearly across the three birth cohort periods, in boys from 0.12% through 0.46% at age 7 years and from 0.18% through 0.54% at age 13 years (Figure 2). In girls, it increased from 0.28% through 0.75% at age 7 years and from 0.25% through 0.67% at age 13 years (Figure 2).

**Figure 3:**
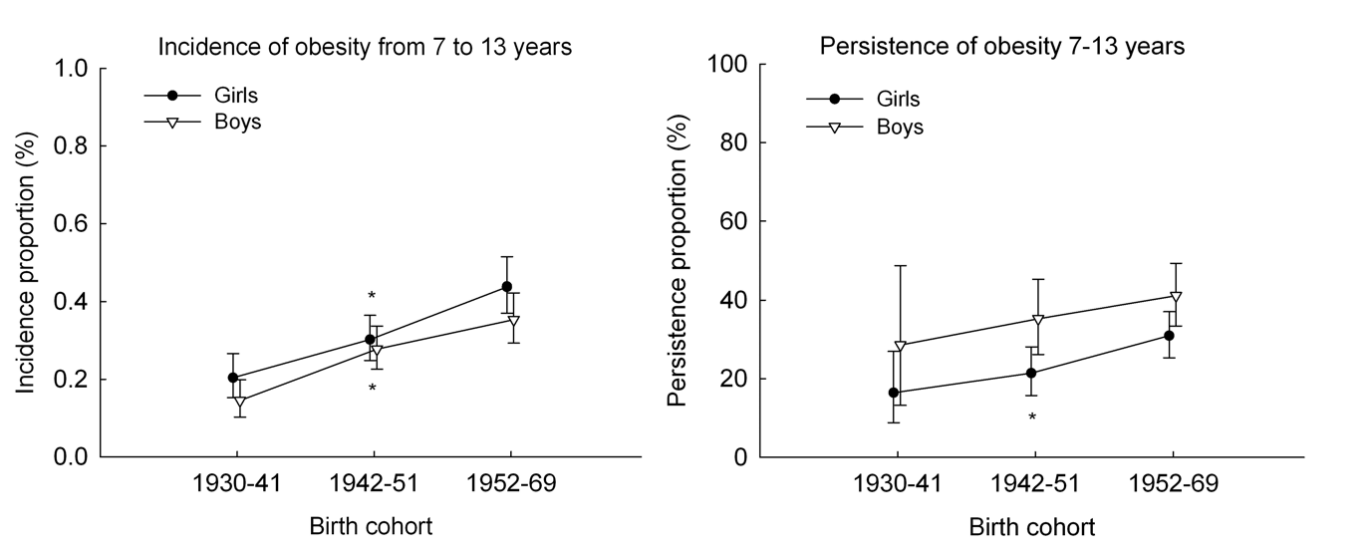
Trends in the incidence and persistence proportion of obesity. The figure shows trends in the incidence (left panel) and persistence (right panel) proportion of obesity (percent with 95% confidence intervals) from age 7 to 13 years by birth cohort (1930–69) among children in the Copenhagen School Health Records Register. Note that in the left panel the range on the y-axis is truncated at 1%. The analysis of incidence is based on 96 558 boys and 95 636 girls who were non-obese at age 7 years. *P* values for non-linearity were 0.41 among boys and 0.63 among girls. The *p* value for linearity was <0.001 among both boys and girls. The analysis of persistence is based on 291 boys and 507 girls who were obese at age 7 years. Obesity was defined by International Obesity Task Force criteria. *P* values for non-linearity were 0.96 among boys and 0.60 among girls. *P* values for linearity were 0.15 among boys and 0.004 among girls. * *p*_linear_ <0.05.

Among *boys*, the incidence proportion of obesity from 7 to 13 years of age increased linearly across the three birth cohort periods from 0.15% to 0.35% (Figure 3). The persistence proportion of obesity tended to increase linearly across birth cohort periods from 28.6% in the 1930–41 birth cohorts to 41.1% in the 1952–69 birth cohorts, although not significantly (Figure 3). Among *girls*, the incidence proportion of obesity from 7 to 13 years increased linearly across the three birth cohort periods from 0.20% to 0.44% (Figure 3). The persistence proportion of obesity increased linearly and significantly (p<0.005) across birth cohorts from 16.4% in the 1930–41 birth cohorts to 31.0% in the 1952–69 birth cohorts (Figure 3).

The complementary remission proportion (1.00 minus the persistence proportion) was 71.4% among boys and 83.6% among girls born in 1930–41. Although it decreased along with the corresponding increase in persistence, it remained high; the remission proportion in the 1952–69 birth cohorts was 59.9% among boys and 69.0% among girls.

**Figure 4:**
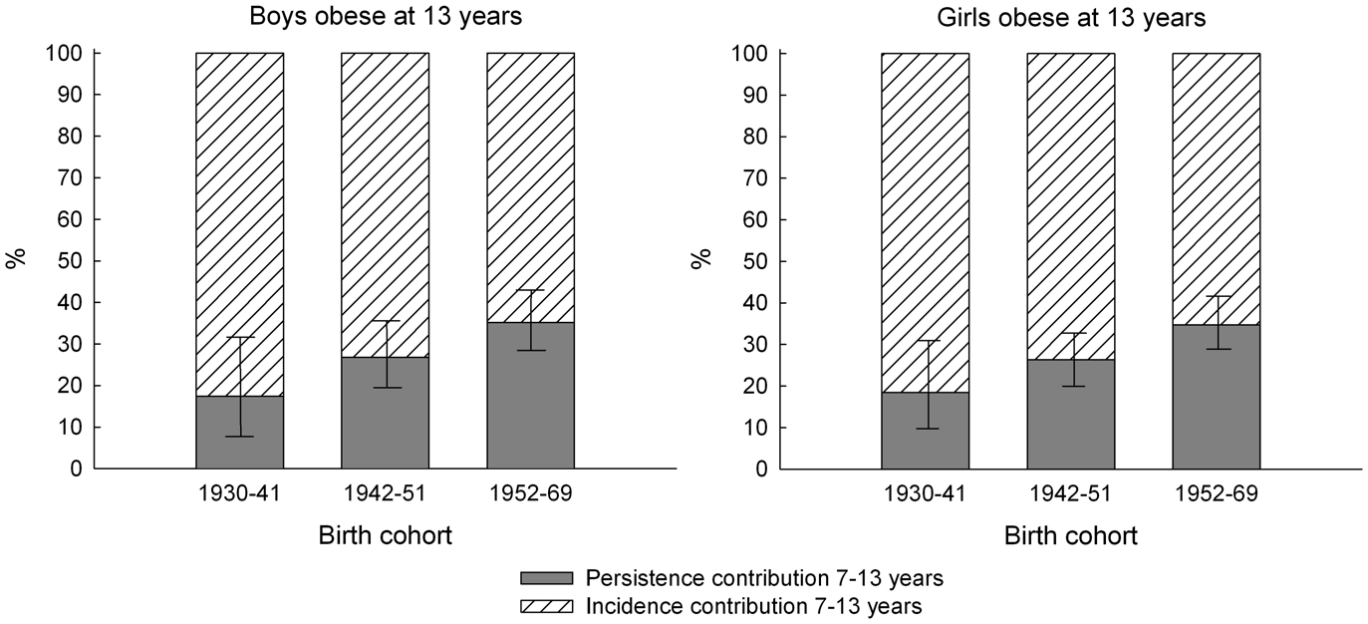
Trends in the incidence and persistence contributions to the prevalence of obesity at 13 years. The figure shows trends in the incidence and persistence contributions (in percent with 95% confidence intervals for the persistence contribution) to the prevalence of obesity by birth cohort (1930–69) among 367 13-year-old obese boys (left panel) and 438 13-year-old obese girls (right panel) in the Copenhagen School Health Records Register. Obesity was defined by International Obesity Task Force criteria. *P* values for non-linearity were 0.92 among boys and 0.97 among girls. *P* values for linearity were 0.01 among boys and 0.006 among girls.

Among those classified as obese at 13 years of age, the proportion of persistently obese children relative to incident obese children since age 7 years increased linearly across the three birth cohorts from 17.4% to 35.1% among boys and from 18.5% to 34.7% among girls (Figure 4).

### BMI at 7 Years and Odds of Obesity at 13 Years of Age

**Figure 5:**
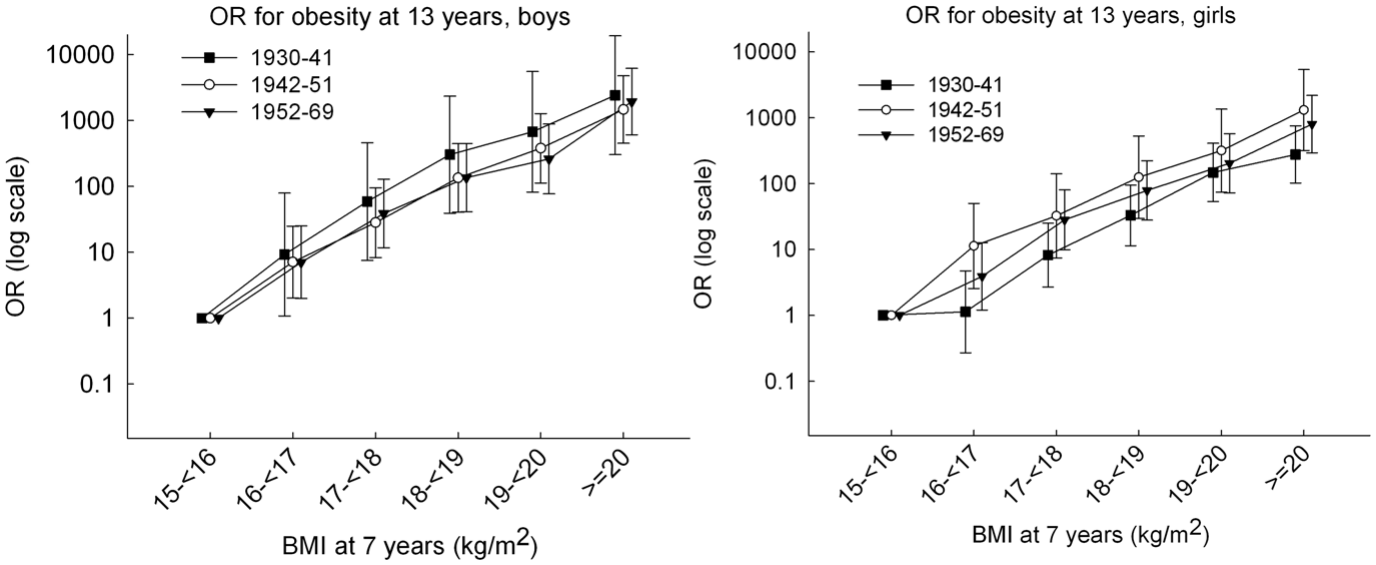
Odds ratio for developing obesity at 13 years by BMI at 7 years and birth cohort. The figure shows the odds ratio with 95% confidence intervals for of obesity at 13 years in relation to BMI at age 7 years and birth cohort (1930–69) among 96 849 boys (left panel) and 96 143 girls (right panel) in the Copenhagen School Health Records Register. Note that the range on the x-axis is truncated at a BMI of 15 kg/m^2^ because no boys and only 5 girls with a BMI below 15 kg/m^2^ at 7 years became obese at 13 years. Obesity was defined by International Obesity Task Force criteria. BMI, body mass index.

The proportion of children who were obese at 13 years increased, as expected, with increasing BMI at 7 years (Figure 1, Table S1 and Table S2). In neither boys or girls was there a significant interaction between BMI at 7 years of age (included as either continuous or categorical variable) and birth cohort on the odds of obesity at age 13 years (*p* values ranged from 0.48 to 0.98). In all three birth cohort periods, the odds of obesity for both boys and girls at 13 years increased approximately linearly on logarithmic scale across the range of BMI at 7 years (Figure 5). Hence, the odds increased exponentially by increasing BMI at 7 years and reached more than 200 for children with a BMI at 7 years above 20 kg/m^2^ (Figure 5).

Assessed in a common logistic regression model, birth cohort and BMI at 7 years were both positively associated with the odds of obesity at 13 years (*p* values ranged from <0.0001 to 0.03). The odds for obesity at age 13 years increased slightly the later the birth cohort; in boys the values were: OR_1942–51 vs. 1930–41_ = 1.31 (95% confidence interval (CI): 0.92, 1.85), OR_1952–69 vs. 1930–41_ = 1.57 (95% CI: 1.12, 2.22), and in girls the values were: OR_1942–51 vs. 1930–41_ = 1.12 (95% CI: 0.82, 1.52), OR_1952–69 vs. 1930–41_ = 1.62 (95% CI: 1.21, 2.16). However, the association between BMI at 7 years and obesity at 13 years was stronger in boys than in girls (*p_interaction_* = 0.006) (Table 2). Nonetheless, within the genders the associations were similar across birth cohorts (Table 2). Per BMI unit increase at age 7 years, the odds of obesity at 13 years increased about 3-fold (Table 2).

**Table 2:** Obesity at 13 years as a function of BMI at 7 years by sex and birth cohort.

			Birth cohort						
	1930–41		1942–51		1952–69		Total		
Sex	OR^a^	95% CI	OR^a^	95% CI	OR^a^	95% CI	OR^b^	95% CI	
**Boys**	3.09	2.66, 3.59	2.93	2.69, 3.19	2.85	2.65, 3.08	2.92	2.77, 3.08	
**Girls**	2.53	2.27, 2.82	2.73	2.52, 2.96	2.64	2.48, 2.83	2.65	2.53, 2.78	

Abbreviations: CI, confidence interval; OR, odds ratio for obesity per BMI unit increase (kg/m^2^).

a Adjusted for age at 7 years in months.

b Adjusted for age at 7 years in months and birth cohort.

## Discussion

In this study we investigated trends in the incidence and persistence of obesity from 7 through 13 years of age during the emergence of the childhood obesity epidemic in Denmark and their relationship with the trend in the prevalence of obesity. Increases in both incidence and persistence contributed to the increasing prevalence. Although decreasing over time, spontaneous remission of obesity occurred in 60% or more of obese children. However, in spite of the increasing prevalence of obesity, the relation between BMI at 7 years throughout its range and the odds of obesity at age 13 were stable over time as defined by the birth cohorts. Obesity at 13 years among children with a BMI at 7 years below the IOTF cut-off point versus those above the cut-off point represents incidence and persistence, respectively. Thus, the results demonstrate that the incidence of obesity increased by birth cohort in all categories of BMI at 7 years among children who were non-obese at 7 years, and that the persistence of obesity increased by birth cohort in all categories of BMI at 7 years among children who were obese at 7 years.

The prevalence of childhood obesity in Denmark increased almost 5-fold during the study period from around 0.1% among the oldest birth cohort to around 0.5% in the youngest of the three birth cohorts [3]. The phases in the increase in obesity prevalence over time were similar for different ages when plotted by year of birth, suggesting that changes in early life initiated these increases in obesity prevalence [3], which was before the economic growth of the nation [6]. The increasing prevalence of obesity at 7 years may be regarded as being due to an increase in the cumulative incidence of obesity from birth to age 7 years. Previous analyses of these data have shown a weak positive relation between birth weight and obesity in school age, but virtually no change in birth weight distribution across the birth cohorts; thus, the rising prevalence of obesity in school age children could not be attributed to increasing birth weight [21].

Our results indicate that the first phase of the obesity epidemic is driven by increases in both a tendency among non-obese children to develop obesity before and during school age and a tendency among children who had developed obesity already by age 7 years to retain the excess weight at the age of 13 years. The median BMI did not change notably meaning that in general children did not become heavier over time, so the increase in obesity is based on increases in the upper tail of the BMI distribution.

BMI, and hence the incidence and persistence rates, may have fluctuated at the different ages between 7 and 13 years. We addressed long-term trends across ages 7 and 13 years as a basis for assessing changes in incidence and persistence independent of short-term variations in body weight. Whether the determinants of initiation and persistence are the same, and whether a select group of children have been exposed or all have been exposed but only a susceptible subgroup was reacting, remains unknown. This study encourages research into the possibilities for modifying the determinants of incidence and persistence of obesity in children.

A review of the genetic component behind longitudinal changes in BMI concluded that the underlying genes are the most important factor contributing to the tracking of BMI [22]. It may be speculated that the children in the upper BMI percentiles have a high degree of genetic predisposition increasing their susceptibility to obesogenic influences that promote both increased incidence and persistence [23]–[26]. A study of pairs of brothers among Swedish draftees found that the genetic variance of BMI increased during the obesity epidemic suggesting that the obesogenic environment has enhanced the influence of adiposity related genes [27].

One possible explanation for the observed trends may be related to factors that also have induced decreasing age at the onset of puberty, which is inversely related to obesity, but the age at onset of puberty declined over time in all BMI groups and therefore does not provide a clue to the increasing degree of obesity in this population [28].

Our study has strengths and limitations. The major strength of this study is that is it based on the CSHRR, which encompasses a very large and unselected prospectively followed population-based cohort with valid anthropometric information from both private and public schools [29]. The long time span covered provided a unique possibility to investigate secular trends in the incidence and persistence of obesity during the first take-off of the childhood obesity epidemic in Denmark. The CSHRR contains information on virtually every school child in Copenhagen, of whom 80% had anthropometric information at both 7 and 13 years. School physicians and nurses measured height and weight with a great attention to detail, and recording remained unchanged during the years included in the CSHRR [5]. Thus, even though the number of obese children was relatively low especially in the older cohorts, any influence of measurement and sampling errors is likely independent of the year of birth. The prevalence of obesity in this study population was much lower than among Danish school-aged children today (around 4%) [30] and than among concurrent school-aged children in other countries (3–20% obese) [31], but it was not much lower than in other countries during the study period (0–2%) [32]–[35]. The relationship between BMI at 7 years and odds of obesity at 13 years was similar across the three phases of the epidemic attesting to the robustness of the association. We find it likely that the results are generalizable to other countries and time periods with different obesogenic environments, and that increases in both incidence and persistence are still involved in the recent development of the epidemic [7].

A number of longitudinal population-based studies based on children born in the years 1978 through 1998 who were measured in the years 1991 through 2004 have consistently reported an increased risk of obese children remaining obese during childhood [11]–[13], [15]–[18]. Few studies have reported the incidence of obesity among children [11]–[14]. In none of these studies has the prevalence of obesity been decomposed into incidence and persistence/remission, and none of them addressed the time trends or the time period of the emergence of the obesity epidemic. In a previous study, conducted on a series of obese and randomly sampled non-obese Danish young men, born 1923 through 1959 and measured around age 20 years in 1943–77, it was found that the BMI during school age was higher for the obese, but unchanged during the emergence of the obesity epidemic [2]. This sort of study design does not allow the distinction between incidence and persistence and was, indeed, the inspiration to launch the formation of the CSHRR and analyze the data as done in the present study.

An important result of this study is that childhood obesity appears to be a more dynamic trait than it is commonly perceived. Although the persistence increased during the study period, spontaneous remission occurred in approximately 60% or more of obese children. In this period - before and during the emergence of the obesity epidemic [3], [6] - efforts to prevent and treat childhood obesity can be assumed to have been non-existent in Denmark. Remission also occurs among contemporary children [11], [12]. The aforementioned analysis of the combined draft board and school health examinations in part of the same study population found that most obese boys did not remain obese in adulthood and that only a few obese adults had been obese throughout school ages [36]. In general, non-overweight children tend to gain body weight and overweight and obese children tend to lose body weight [37]. Considering that the effect of current interventions to prevent and treat childhood obesity is limited [38], [39], the finding of spontaneous remission is encouraging although the tendency has declined along with the development of the epidemic. Thus, obesity may not be a chronic disease among all children and it remains important to predict who may and may not be persistently obese and elucidate why.

The increased incidence and persistence of obesity are of concern because of the potential negative immediate and long-term health consequences [29], [40]. However, a recent Finnish study showed that the increased cardiovascular risk by childhood obesity pertained only to obese children who were obese also as adults, whereas it had disappeared among obese children who had become non-obese in adulthood [41]. It may be speculated that the increased prevalence seen in lifestyle-related disease among children [40] is due to an even higher persistence of obesity among today’s children, and thus increasing cumulative exposure to excess weight over the lifetime of those from more recent birth cohorts [42].

In conclusion, the increased prevalence of obesity at age 13 years among boys and girls in the emerging phase of the epidemic in Denmark can be ascribed to both an increased incidence before and after age 7 years and an increased persistence from 7 through 13 years of age. The results from this study suggest that the factors leading to the increased prevalence of obesity may be both obesity-initiating factors and factors that increase the persistence of obesity; both deserve attention in future studies addressing the causality of obesity. Finally, the results showed that spontaneous remission from obesity occurred in an appreciable proportion of children who were obese at 7 years of age despite of the emergence of the obesity epidemic.

## Supporting Information

Table S1 **Number of boys included in the study by BMI at age 7 and 13 years.**
(PDF)

Table S2 **Number of girls included in the study by BMI at age 7 and 13 years.**
(PDF)
