# Supplementary material for: Contributions of Incidence and Persistence to the Prevalence of Childhood Obesity during the Emerging Epidemic in Denmark
Source: PLoS One. 2012 Aug 10;7(8):e42521. doi: 10.1371/journal.pone.0042521 (PMC3416857; doi:10.1371/journal.pone.0042521)
Supplement: Table S1 — Number of boys included in the study by BMI at age 7 and 13 years. (PDF) [file pone.0042521.s001.pdf]

**Table S1**. Number of boys included in the study by BMI at age 7 and 13 years.

| Boys (N) | BMI at 13 years (kg/m$^2$) | | | | | | | | | Total | Obese by IOTF criteria at 7 years |
|---|---|---|---|---|---|---|---|---|---|---|---|
| **BMI at 7 years (kg/m$^2$)** | **<16** | **16-<18** | **18-<20** | **20-<22** | **22-<24** | **24-<26** | **26->28** | **28->30** | **>=30** | | |
| **<14** | 3,951 | 2,665 | 400 | 56 | 12 | 6 | 0 | 0 | 0 | **7,090** | 0 |
| **14-<15** | 4,943 | 15,048 | 4,690 | 667 | 104 | 19 | 2 | 0 | 0 | **25,473** | 0 |
| **15-<16** | 1,009 | 15,493 | 13,501 | 2,943 | 506 | 113 | 16 | 3 | 1 | **33,585** | 0 |
| **16-<17** | 59 | 3,601 | 10,074 | 4,690 | 1,032 | 256 | 61 | 14 | 1 | **19,788** | 0 |
| **17-<18** | 12 | 337 | 2,718 | 2,823 | 1,056 | 306 | 92 | 19 | 7 | **7,370** | 0 |
| **18-<19** | 2 | 37 | 358 | 801 | 637 | 267 | 101 | 27 | 4 | **2,234** | 0 |
| **19-<20** | 0 | 5 | 49 | 185 | 225 | 151 | 63 | 26 | 8 | **712** | 0 |
| **20<21** | 0 | 2 | 12 | 44 | 91 | 90 | 56 | 21 | 9 | **325** | 30 |
| **21<22** | 0 | 0 | 4 | 14 | 31 | 30 | 33 | 18 | 11 | **141** | 130 |
| **>=22** | 0 | 0 | 1 | 6 | 18 | 28 | 27 | 20 | 31 | **131** | 131 |
| **Total** | **9,976** | **37,188** | **31,807** | **12,229** | **3,712** | **1,266** | **451** | **148** | **72** | **96,849** | |
| **Obese by IOTF criteria at 13 years** | 0 | 0 | 0 | 0 | 0 | 0 | 149 | 148 | 72 | | |
