# Supplementary material for: Contributions of Incidence and Persistence to the Prevalence of Childhood Obesity during the Emerging Epidemic in Denmark
Source: PLoS One. 2012 Aug 10;7(8):e42521. doi: 10.1371/journal.pone.0042521 (PMC3416857; doi:10.1371/journal.pone.0042521)
Supplement: Table S2 — Number of girls included in the study by BMI at age 7 and 13 years. (PDF) [file pone.0042521.s002.pdf]

Table S2. Number of girls included in the study by BMI at age 7 and 13 years.

**Girls (N)**	**BMI at 13 years (kg/m^2)**									**Total**	**Obese by IOTF criteria at 7 years**
**BMI at 7 years (kg/m^2)**	**<16**	**16-<18**	**18-<20**	**20-<22**	**22-<24**	**24-<26**	**26->28**	**28->30**	**>=30**		
**<14**	4,389	5,331	1,504	214	40	4	3	1	0	**11,486**	0
**14-<15**	2,807	12,937	8,536	1,997	290	48	11	2	0	**26,628**	0
**15-<16**	553	8,313	13,078	5,643	1,225	202	39	9	1	**29,063**	0
**16-<17**	52	1,676	6,651	5,721	1,946	504	89	18	8	**16,665**	0
**17-<18**	9	227	1,644	2,802	1,659	581	157	46	13	**7,138**	0
**18-<19**	5	43	348	974	915	480	177	45	26	**3,013**	0
**19-<20**	0	10	76	233	362	260	139	47	28	**1,155**	0
**20<21**	0	2	21	67	148	158	110	52	28	**586**	100
**21<22**	0	0	6	23	49	64	47	28	23	**240**	238
**>=22**	0	0	1	9	22	28	44	24	41	**169**	169
**Total**	**7,815**	**28,539**	**31,865**	**17,683**	**6,656**	**2,329**	**816**	**272**	**168**	**96,143**	
**Obese by IOTF criteria at 13 years**	0	0	0	0	0	0	26	244	168		
